# A Survey on Potentially Beneficial and Hazardous Bioactive Compounds in Cocoa Powder Samples Sourced from the European Market

**DOI:** 10.3390/foods13152457

**Published:** 2024-08-03

**Authors:** Luigi Esposito, Matteo Perillo, Carla Daniela Di Mattia, Annalisa Scroccarello, Flavio Della Pelle, Dario Compagnone, Giampiero Sacchetti, Dino Mastrocola, Maria Martuscelli

**Affiliations:** 1 Department of Bioscience and Technology for Food, Agriculture and Environment, University of Teramo, Via R. Balzarini 1, 64100 Teramo, Italy; lesposito2@unite.it (L.E.); cdimattia@unite.it (C.D.D.M.); ascroccarello@unite.it (A.S.); fdellapelle@unite.it (F.D.P.); dcompagnone@unite.it (D.C.); gsacchetti@unite.it (G.S.); dmastrocola@unite.it (D.M.); 2 Department of Biomedical and Neuromotor Sciences, University of Bologna, Via Massarenti 9, 40138 Bologna, Italy; matteo.perillo2@unibo.it

**Keywords:** biogenic amines, polyphenols, salsolinol, organic processing, raw cocoa, alkalization treatment

## Abstract

Cocoa (*Theobroma cacao*, L.) represents an important market that gained relevance and became an esteemed commodity thanks to cocoa powder, chocolate, and other related products. This work analyzed 59 cocoa powder samples from the European market. Three distinct subgroups were identified: organic or conventional, alkalized or not alkalized, and raw or roasted processing. The impact of the technological process on their pH, color, and compositional traits, as well as their content of biogenic amines and salsolinol, was evaluated. The phenolic fraction was also investigated through both common and emerging methods. The results depict that the influence of the agronomical practices (organic/conventional) did not significantly (*p* < 0.05) affect the composition of the cocoa powders; similarly, the roasting process was not a determinant of the compounds traced. On the other hand, the alkalinization process greatly impacted color and pH, no matter the cocoa’s provenience or obtention or other processes, also resulting in reducing the phenolic fraction of the treated samples. Principal component analysis confirmed that the alkali process acts on pH, color, and phenolic composition but not on the content of other bioactive molecules (biogenic amines and salsolinol). All the samples were safe, while the alkalized powders saw a great reduction in beneficial biocompounds. A novel strategy could be to emphasize on the label whether cocoa powder is non-alkalized to meet the demand for more beneficial products.

## 1. Introduction

Cocoa powder is consumed and appreciated worldwide, not only as a key ingredient in beverages but also in various products. Even in small amounts, it significantly affects color, flavor, and functionality due to its rich bioactive compounds, which have antioxidant and anti-inflammatory properties [1]. Recently, Tuenter et al. [2] introduced the mood pyramid concept, emphasizing the impact of cocoa’s bioactive molecules on mood and cognition. Cocoa’s health benefits stem from its complex composition and intense processing, involving biogenic amines, polyphenols, methylxanthines, and their interactions [3]. Biogenic amines (BAs) in cocoa, such as polyamines, histamine, tyramine, dopamine, serotonin, and salsolinol, can naturally occur or form during processing through microbial/oxidative decarboxylation, amination, transamination, thermal decarboxylation, or lipid peroxidation [4,5,6]. While some BAs are beneficial at low levels, high intake or their interaction with drugs, alcohol, or polyamines can be harmful [7,8]. Researchers are exploring strategies to reduce BAs in foods, although this is challenging, especially in fermented products. The regulatory limits for BAs, except for histamine in fish, are not well defined, making it crucial to monitor BAs in cocoa and other foods [9,10,11]. Tetrahydroisoquinoline compounds, naturally occurring in plants and animals, have gained interest due to their bioactivity. Salsolinol, a notable tetrahydroisoquinoline, binds to dopamine receptors and may contribute to chocolate addiction [12,13,14,15].

Cocoa polyphenols, especially flavanols like epicatechin and catechin, are abundant in fresh cocoa beans (2% *w*/*w*) and contribute to cocoa’s health benefits. These include phenolic acids, stilbenes, and N-phenylpropenoyl-L-amino acids (NPAs) [16,17,18,19]. Methylxanthines, derived from xanthine, also impact the taste and bioactivity of cocoa products. Cocoa’s bioactive composition varies with genotype, origin, cultivation, and postharvest conditions [10]. Processing steps like fermentation, drying, roasting, and alkalization (the Dutch process) affect these compounds. Alkalization enhances flavor and solubility but can reduce phenolic content and antioxidant capacity, impacting nutritional value [20,21,22].

While the concentration of phenolic compounds and the impact of processing have been intensely researched, few studies have focused on BAs and polyphenols in cocoa powder. This study surveyed 59 commercial cocoa powder samples to assess their quality and safety, evaluating color, pH, total polyphenolic content, antioxidant activity, and BA levels. It also examined how the processing conditions affected the bioactive compounds in different sample subclasses [1].

## 2. Materials and Methods

### 2.1. Origin of the Samples

Fifty-nine commercial samples of cocoa powder were collected directly from the European market; the origin of the samples was related to the largest eight providers (85%), followed by other minor providers (15%), reflecting the distribution of international production on the market. Providers were identified with an alphabetical code (Table 1), and each sample was assigned an identification numeric code; the geographical origin of the raw cocoa beans has been reported in Table 1. This table also lists information about the technological treatment of each investigated sample; a total of 59 cocoa powder samples were analyzed, of which 18 were commercially labeled as “organic” and 9 were claimed to be “raw” (obtained from unroasted cocoa beans), while 29 samples were alkalized (as declared by the providers).

### 2.2. Color Analysis

Color analysis of the cocoa samples was carried out using a Minolta bench-top colorimeter CR-5 Spectrally based (Konica Minolta, Tokyo, Japan). Before the analysis, two calibrations were carried out, one with a black standard and the other one with a white standard. For each measurement, a single layer (4–5 mm) of cocoa powder was spread onto a Petri dish. The analysis was repeated five times on each sample. The following color coordinates were determined: lightness (L*), red/green (+/−) coordinate (a*), (+/−) yellow/blue coordinate (b*), and hue angle (h°)= [arctang (b*/a*).] The a*/b* ratio was calculated to determine the red notes in the cocoa powder samples. The color difference [23] between the set of non-alkalized cocoa powder samples (control) and alkalized ones was calculated as follows:

∆E_NA_ = (∆L*^2^ + ∆a*^2^ + ∆b*^2^)^0.5^

### 2.3. Moisture and pH Determination

The pH of the defatted cocoa powder was measured by diluting it in distilled water (1:1) after 1 h of rest by using an electrode probe connected to a pH meter (Mettler Toledo, FE20, Columbus, OH, USA).

Moisture content was determined according to the AOAC official procedures [24]. In particular, 1 g of the sample was dried in a force -air-drying oven at 105 °C to a constant weight.

### 2.4. Fat Content Determination

The cocoa powder samples were defatted by hexane washing according to the AOAC official methods, 2012 [24]. In brief, to 4 g of the sample, 25 mL of hexane was added; the mixture was then vortexed for 1 min and centrifuged (5000× *g* for 10 min) with an ALC4237R refrigerated centrifuge (ALC Intl., Cologno Monzese, Italy), with the supernatant discharged each time. The defatting process was performed three times; eventually, to completely remove the hexane from the sample, the lipid-free cocoa powder was air-dried at room temperature. The total fat content was estimated by calculating the residual sample weight with respect to the starting weight.

### 2.5. Extraction of the Phenolic Fraction

All the reagents and solvents employed for the assays were of analytical grade and were purchased from Sigma-Aldrich (St. Louis, MO, USA).

#### 2.5.1. Polyphenol Conventional Liquid–Liquid Extraction

The defatted samples were further ground with a mortar and pestle to reduce the powder size and allow for better contact of the extracting solvent with the sample. The sample extraction was carried out according to the AOAC official methods, 2012 [24], with some modifications. One gram of the defatted sample was added to 5 mL of 70:29.5:0.5 acetone/water/acetic acid; the mixture was vortexed for 1 min, then sonicated in an ultrasonic bath at 20 °C for 10 min, and finally centrifuged (10,000× *g* for 10 min). The supernatant was recovered and filtrated through cellulose filters. The extracted polyphenols were then stored in the freezer at −20 °C until the analyses.

#### 2.5.2. Dimethylsulfoxide-Based Polyphenol Fast Extraction

A total of 0.1 g of cocoa powder, without any pretreatment, was solubilized in 1.5 mL of DMSO, vortexed for 1 min, and sonicated in an ultrasonic bath for 5 min at 20 °C, according to the strategy proposed by Della Pelle et al. [25], with some modifications. The dispersion was centrifugated at 10,000× *g* for 5 min, and the supernatant was recovered and stored at −20 °C in the dark.

### 2.6. Phenolic Compound Evaluation

#### 2.6.1. The Folin–Ciocalteu Assay

The Folin–Ciocalteu assay was executed according to Di Mattia et al., 2015 [26], with slight modifications. A total of 20 μL of the properly diluted cocoa sample, extracted in the conventional way according to Section 2.5.1, was added to 20 μL of Folin–Ciocalteu reagent and orbitally stirred for 3 min with an orbital shaker (SSL1, Stuart equipment, Belfast, UK). Then, 400 μL of sodium carbonate (7.5% Na_2_CO_3_) and deionized water was added up to a final volume of 1000 μL. The solution was stirred at room temperature for 60 min in the dark, and the total polyphenol content was determined at 760 nm; the absorbance values were recorded using a JENWAY 6400 spectrophotometer from Barloworld Scientific (Staffordshire, UK). Gallic acid was employed as a reference standard to calibrate the method.

#### 2.6.2. The AuNP-Based Assay

A gold nanoparticle (AuNP)-based colorimetric assay was performed according to Della Pelle et al., 2015 [27], to evaluate the samples’ reducing capacity. The assay was conducted in 1.5 mL obscured microcentrifuge tubes; the cocoa samples extracted according to Section 2.5.1 were employed for the analysis, which were diluted in DMSO prior to analysis. Thus, 30 μL of the appropriately diluted cocoa extract was mixed with 210 μL of DMSO and stirred for 1 min at 300 rpm with the orbital shaker. Then, 25 μL of 2.0 × 10^−2^ mol L^−1^ HAuCl_4_ and 235 μL of 1.0 × 10^−2^ mol L^−1^ phosphate buffer (PB), pH 8.0, was added. Afterward, the solution was mixed for 1 min at 300 rpm with an orbital shaker and left to react for 5 min at 45 °C in a water bath. Finally, the reaction was blocked at −20 °C for 5 min, and the absorbance intensity, ascribed to the formation of the AuNPs, was recorded at 540 nm against the blank (the reaction mix without the sample). Gallic acid was employed as a reference standard to calibrate the method.

#### 2.6.3. Electrochemical Measurement of Catechins

According to the method proposed by Della Pelle et al., 2019 [28], the antioxidant activity, on the basis of the charge of flavan-3-ol structures, was evaluated using an electrochemical method. Thus, a screen-printed electrode (with a three-electrode configuration: working and counter electrodes of graphite and silver as the pseudo-reference electrode, from EcoBioServices, Florence, Italy), modified with carbon black N220 from Cabot Corporation (Ravenna, Italy) and molybdenum disulfide from Sigma-Aldrich (St. Louis, MO, USA), according to Della Pelle et al., 2019 [28], was employed; from this point forward, the electrochemical sensor will be referred to as the nanostructured sensor (nSensor). The cocoa samples extracted according to Section 2.5.2 were diluted in 0.1 mmol L^−1^ PB, pH 7.0, to fit the dynamic range, and 100 µL of the samples was used to perform the analysis. Different pulse voltammetry (DPV), performed with a portable Palmsens 4 potentiostat (Palm Instruments BV, Houten, the Netherlands), was employed for measurement in a potential window from −0.15 to 0.35 V and with a pulse width of 50 ms, a pulse amplitude of 20 mV, and a scan rate of 50 mV s^−1^. Before use and to regenerate the electrode, 5 consecutive cycles of cyclic voltammetry were conducted in 0.1 mmol L^−1^ PB, pH 7, and 0.1 mol L^−1^ KCl and in a potential range from −0.30 V to +0.7 V (vs. pseudo-Ag/AgCl) at a scan rate of 0.5 V s^−1^. Catechin was employed to calibrate the method, but the data were reported as gallic acid equivalents (GAeq) using an experimental correction factor, obtained according to the ratio “gallic acid calibration slope/catechin calibration slope” [27,28].

### 2.7. Biogenic Amine Determination

The defatted samples were subjected to BA extraction, detection, identification, and quantification by HPLC, optimizing the method described by Delgado-Ospina et al. [10]. All the chemicals were of analytical reagent grade and supplied by Carlo Erba (Milan, Italy). The standards were obtained from Sigma (Bellefonte, PA, USA).

In short, 1.0 g of sample was added to 5.0 mL of 0.1 N HCl and stirred in a vortex (1 min) and an ultrasound (20 min). It was centrifuged at a relative centrifugal force of 4472× *g* for 10 min (the refrigerated centrifuge NEYA 16r, Mumbai, India), and the supernatant was recovered. Then, 150 µL of saturated NaHCO_3_ was added to 0.5 mL of the supernatant, adjusting the pH to 11.5 with 0.1 N NaOH. For derivatization, 2.0 mL of dansyl chloride/acetone (10 mg mL^−1^) was added, and it was incubated at 40 °C for 1 h under agitation (195 stokes) (Dubnoff Bath-BSD/D, International PBI, Milano. Italy). To remove excess dansyl chloride, 200 µL of 30% ammonia was added, allowed to stand for 30 min at room temperature, and diluted with 1950 µL of acetonitrile.

In a Spherisorb S30ODS Waters C18-2 column (3 µm, 150 mm × 4.6 mm ID), 10 µL of sample was injected with gradient elution of acetonitrile (solvent A) and water (solvent B) as follows: 0–1 min, 35% B, isocratic; 1–5 min, 35–20% B, linear; 5–6 min, 20–10% B, linear; 6–15 min, 10% B, isocratic; 15–18 min, 35% B, linear; 18–20 min, 35% B, isocratic. The identification and quantification of cadaverine (CAD), dopamine (DOP), ethylamine (ETH), histamine (HIS), 2-phenylethylamine (PHE), putrescine (PUT), salsolinol (SAL), serotonin (SER), spermidine (SPD), spermine (SPM), and tyramine (TYR) was performed by comparing the retention times and using the calibration curves of the pure standards, respectively. The results are reported as mg of BA kg^−1^ of defatted dry weight (of DDW).

### 2.8. Statistical Analyses

All determinations were carried out in triplicate, except where differently indicated.

The means and standard deviations for each numeric variable in the sample were computed. Differences in the mean values between organic and non-organic, raw and non-raw, and alkalized and non-alkalized cocoa samples were assessed through a t-test, but when the variable distribution was normal on the basis of the Shapiro–Wilk test, in other cases, the Mann–Whitney (MW) test [29] was used. Since in some cases the sample size was limited (e.g., the raw and organic samples), the Monte Carlo method with 1 × 10^6^ simulation was applied to both the Mann–Whitney test and the *t*-test in order to generate random replicate values that closely approximated the distribution of samples that would likely be collected in a broader survey. All statistics were calculated using XLSTAT 2021 software (Addinsoft, New York, NY, USA/Paris, France).

In addition, principal component analysis (PCA) [30] was carried out to obtain a representation of the numeric variables (excluding “total biogenic amines”) in a space of reduced dimensionality. The dimensionality of such space, i.e., the number of principal components (PCs) to retain in the analysis, was defined through the elbow method. A score plot of the first 2 PCs was produced to visualize the distribution of the cocoa samples in the most meaningful bidimensional space. PCA was performed using R Statistical Software (v4.2.2) [31].

## 3. Results and Discussion

An extensive survey was carried out on 59 cocoa powder samples from the European market. The cocoa samples were segmented into distinct subgroups: organic/conventional, according to the agronomic techniques used for their production; alkalized/not alkalized, according to the alkalinization process used prior to roasting; and raw/not raw, according to adoption of the roasting process (or not). In particular, the “organic” mark and the keyword “raw” were present on the labels since they distinguish two types of commercial categories of cocoa powder.

### 3.1. Color Indices and pH

The color parameters and pH, reported in Table 2, were used as technological indices of the roasting process and alkalinization, respectively.

No differences in pH or color between the organic and conventional samples were observed since many factors, such as the fermentation process, roasting, and alkalinization, can largely influence cocoa and cocoa powder’s color and pH [10,26].

As expected, alkalization increased the pH of the cocoa powder (Table 2). According to the classification of alkalized cocoa powder [32], all of the investigated commercial samples fell into the medium-alkalized range (pH 6.5–7.2), whereas no samples were assigned into the highly alkalized range (pH > 7.6). The pH of the non-alkalized samples fell within the range of natural cocoa powder (pH 5.3–5.8).

The alkalized samples also showed lower luminosity (L*) and hue angle (h°) values, indicating a higher extent of browning. It is known that Maillard condensation is favored in a basic environment since nucleophilic amino nitrogen groups are not protonated [33]. Moreover, recent studies have reported on the relationship between the generation of polar and non-polar chromophores and the chemical arrangement of flavan-3-ols(+)catechin or epicatechin, which occur in cocoa matter during the alkalization process [34,35]. The color difference between the alkalized and non-alkalized samples as calculated by the ΔE_NA_ index is about 1.5, which is below the 2.3 threshold identified for a just-noticeable difference to the human eye [36].

The raw samples showed lower L* values than the roasted ones, with an average difference of about 6 points. Regardless, differences in cocoa in terms of the chemical composition, pH, and moisture content can largely affect the extent of the browning reaction. The pH values of the unroasted samples were lower than those of the roasted ones since they (with the exception of sample 53) were not alkalized.

### 3.2. Fat and Moisture

The moisture content for each sample was measured, and constant values were found (2% ± 0.07), so this was considered equal for all the powders. The total fat content was estimated via gravimetry (Section 2.4); no significant trends were observed for the different cocoa powder classes. On the other hand, a heterogeneous fat content, ranging from 5.6% to 27.3%, was recorded (Table 3).

### 3.3. Polyphenol Evaluation

The phenolic fraction of the cocoa samples was characterized by using classic and emerging spectrophotometric methods, i.e., the Folin–Ciocalteu (Folin) and gold nanoparticle-based (AuNP) assays. The phenolic fraction was also estimated using an electrochemical nanostructured sensor (nSensor) developed by Della Pelle et al., 2019 [28]. This sensor allows for the selective determination of total flavonoids, including their polymers. For all methods, the detailed procedures are reported in the Materials and Methods section.

The Folin assay returns information concerning the total phenolic content and relies on the ability of phenolic compounds to quantitatively reduce the Folin reagent, whereas the AuNP-based assay evaluates the total phenolic content (TPC) on the basis of the phenolic compounds’ intrinsic antioxidant capacity. Indeed, the AuNP assay’s principle relies on the PCs’ ability to reduce gold cations Au(III) into the metal form Au (0) and stabilize them in the form of colloidal nanoparticles (AuNPs) [27]; the latter give rise to an optical response. In this case, the phenolic structure plays a key role in the AuNPs’ formation due to the resulting antioxidant capacity and stabilizing ability; for these reasons, the method returns information on the TPC’s intrinsic reactivity [37]. Despite the different principles, the Folin and AuNP assays return similar data; a slight overestimation for the Folin assay was observed since Folin reagent is not selective, and the overestimation could be attributed to other reducing species; on the other hand, the AuNP assay is more influenced by phenolic compounds’ intrinsic antioxidant capacity. Nevertheless, high correlation between the two methods was confirmed by the high Pearson’s coefficient (R = 0.95) and the correlation equation’s slope being close to 1 (y = 1.0647x − 4.3022), indicating the numerical match of the data. The strong correlation observed is remarkable due to the different PC extraction procedures performed before the assay, i.e., conventional extraction for the Folin assay (see Section 2.5.1) and direct phenolic compound solubilization from the cocoa powder using DMSO for the AuNP-based assay (see Section 2.5.2); these data confirm that the DMSO-based free extraction approach is a promising strategy for straightforwardly and rapidly determining the PCs in fat-rich samples.

Overall, the same trend in the TPC was pointed out by the two methods; however, a lower TPC was reported for the alkalinized cocoa powders compared to the non-alkalinized samples, whereas a higher TPC was observed for the raw cocoa powder (Table 3). No significant differences were highlighted between the organic and non-organic samples.

On the other hand, the nSensor-based method allowed us to selectively estimate the flavonoids and their polymers. This method relies on the flavonoids’ ability to donate electrons at the sensor’s working electrode’s surface under an applied potential which promotes the selective oxidation of these structures [28]. Even in this case, the sensor allowed us to evaluate the electron donor ability of flavonoids; the latter is intrinsically influenced by their antioxidant capacity. As expected, there are fewer nSensor data than those from the two spectrophotometric assays, about half compared to the AuNP assay and one-third compared to the Folin assay, due to the method’s selectivity (Table 1 and Table 3). Despite this, the flavonoid content is consistent with the trends in the TPC observed for the Folin and AuNP assays. As observed based on the data, a higher correlation with the AuNP test was found (R = 0.97) than with the Folin method (R = 0.95), confirming that the nSensor, in addition to its selectivity, returns information more closely related to the antioxidant capacity of the studied phenolic structures.

The TPC values of the samples covered a quite broad interval of contents (Table 3), ranging from 3.63 to 140.89 mg_GAE_ g^−1^, giving an indication of the wide variability in the total content of phenolic compounds in cocoa powder, which, in turn, is related to the variability of the raw materials, as well as that of the processing conditions adopted. Such a wide range comprises the values reported in other studies [23,32,38].

For this reason, as carried out for the color parameters and pH values (Table 2), the samples’ total phenolic content values, as evaluated by the Folin and nSensor-based methods, were also segmented according to the technology of production (organic vs. conventional) and processing (alkalinization and roasting) and the results reported in Table 4; in order to evaluate the effect of the technology of production and processing on the phenolic content, the latter were calculated on a defatted dry basis.

A significant impact on both the Folin and nSensor values was observed as a consequence of the roasting and alkalization processes, while no significant impact was found as a consequence of different agronomic practices.

Specifically, the raw and non-alkalized cocoa powders were characterized by higher contents of total polyphenols and flavanols (nSensor) than the roasted and alkalized ones, as both roasting and alkalization processes are indeed reported to significantly impair phenolic compounds and their bioactivity [22].

According to the literature, during roasting, a decrease in the TPC ranging from 28% to more than 50% can be found depending on the processing temperature adopted [39], and this has been related to the oxidation of flavanols and proanthocyanidins. On average, in the samples under study as well, the reduction in both the TPC and nSensor values in the roasted samples accounted for about 50% when compared with the values for the raw cocoa powders.

The chemical oxidation of polyphenols, which takes place during roasting, induces polymerization reactions which are responsible for browning [40]. The energy of activation of polyphenol oxidation (between 60 and 80 kJ mol^−1^) is lower than that of melanoidin formation (132 kJ mol^−1^); thus, the browning observed during roasting is not solely dependent on the occurrence of a Maillard reaction but also polyphenol oxidation, with a consequential effect on the color parameters (Table 2).

As far as alkalization is concerned, it has been proven to strongly affect the content of phenolic compounds, generally causing a reduction, which is strictly related to the degree of alkalization when all the other conditions are equal. In fact, losses in the content of total polyphenols of around 27%, 54%, and 63% are reported for lightly, medium-, and heavily alkalinized cocoa powder, respectively [32]. In the samples under investigation in the present study, the reduction in both the TPC and flavanol (nSensor) values in the alkalized samples accounted, on average, for about 50% and 60%, respectively, when compared with the non-alkalized cocoa powders. Similar percentages of reduction (45.5%) in the content of total polyphenols were also reported in a work by Todorovic et al., 2017 [41], where 11 cocoa powder samples (6 alkalinized and 5 non-alkalinized) were considered.

### 3.4. Biogenic Amines

As known, biogenic amines (BAs) are ubiquitous compounds found at high contents in fermented products. Cocoa and its derivatives may contain variable quantities of BAs with respect to several characteristics. Mainly, technological processes are responsible for their accumulation in foods, but even agricultural practices, geographical origins, and species varieties can have a direct influence on their final content [10].

Many studies have seen the evolution of BAs in cocoa seeds during fermentation and roasting [26,42,43].

The present research shows that most of the cocoa samples sourced from the European market contain very low levels of biogenic amines. As shown in Table 5, the median result was 0.00 mg kg^−1^_DDW_ for all the biogenic amines, demonstrating that their occurrence is quite random and would appear to be unrelated to a specific variable related to processing or chemical characteristics. The peculiar distribution of the BAs even in fermented products such as cocoa powders or cocoa-based products is also influenced by thermal treatments, which have been found to induce the chemical degradation of amino acids even after the fermentation step [42]. The data obtained in this study generally confirm the existing ranges of BAs identified in cocoa powders or in fermented cocoa beans (roasted and raw), hardly ever being hazardous to the healthy population [42].

BAs were detected in 31% of the samples, with a median value (relative to positive BA samples) of 154.08 mg kg^−1^_DDW_, in a range of 0.00–480.87 mg kg^−1^_DDW_ (Table 3).

The ability of the intake of some foods to trigger migraine episodes, particularly cocoa products, may be due to BAs such as tyramine or phenylethylamine [44]; in our study, neither TIR nor PHE were found in the cocoa powder. Moreover, CAD and PUT were also not detectable in the samples investigated.

Most of the samples were found to contain SER and HIS (22% and 19% positive, respectively). In any case, it is important to highlight that the highest HIS level (139.28 mg kg^−1^_DDW_) was found in a sample derived from conventionally grown cocoa, with roasted cocoa beans, and processed by alkalization (sample 34). High levels of HIS may lead to hypotension, nausea, migraine, abdominal pain, and heart problems [7]. No observed adverse effect level (NOAEL) was observed after exposure to 50 mg of histamine per person per meal in healthy individuals; this would be hard to reach, even by eating 2.5 g of cocoa powder, which is the recommended dose according to EFSA 2012 [45]. Moreover, the adverse role of HIS (and TYR) on human health is enhanced in sensitive individuals (with histamine intolerance) and potentiated by the intake of alcohol and some drugs that have anti-depressant and anti-hypertensive effects [8]. It is also important to highlight that amines may be increased after gastrointestinal digestion, according to a recent study [46]. These authors tested the bioaccessibility of biogenic amines in cocoa dark chocolate through in vitro simulation of oral, gastric, and intestinal digestion. High bioaccessibility with the slight influence of digestive enzymes was found for all amines. In vitro digestion showed that pepsin increased the accessibility of polyamines, while pancreatin positively acted on HIS and CAD accessibility.

The cocoa powder samples were rich in SER and DOP, with their results at very high levels, confirming the data from the literature [6,47,48]. Dopamine and serotonin are neurotransmitters that play an important role in the brain’s reward system. DOP is involved in motivation, the reinforcement of behaviors, and pleasure; SER is a calming neurotransmitter. In the brains of animal organisms, endogenous dopamine and serotonin levels are increased by many different types of substances, including dark chocolate. Most of the literature argues that dopamine or serotonin synthesis is an essential way in which an organism can activate positive behavior [49,50]: SER synthesis has been correlated with the presence of its precursors (e.g., tryptophan), and phenylethylamine is a precursor of DOP. McCutcheon evidenced that dopamine is sensitive to the nutritional value of certain foods [51]. Flavonoids, contained in dark chocolate, also seem to stimulate dopamine synthesis in brain [52]. The study in question was conducted on rats, and more research is needed to confirm these findings in humans.

In light of all this speculation, the role of exogenous DOP and SER should be better understood in order to correctly highlight the role that food rich in these compounds can have and whether its intake really has a positive effect neurologically on mood and behavioral aspects.

The literature reports that cocoa and chocolate contain the tetrahydroisoquinoline alkaloid salsolinol up to a concentration of 25 mg/g; taking the detected concentration and its pharmacological properties into account, salsolinol seems to be one of the main psychoactive compounds present in cocoa and chocolate and might be part of chocolate addiction [15]. To the best of our knowledge, this is the first study reporting analyses of the salsolinol content in cocoa powder. In the investigated samples, SAL was only detected in a few samples (7%), and as it is possible to see in Table 5, its maximum content was less than 2 mg kg^−1^_DDW_, in a non-organic, roasted, and non-alkalized sample (N° 33). In any case, the presence of salsolinol in food could be interpreted as unhealthy because it can act as a neurotoxin which kills dopaminergic neurons [53]. High concentrations of salsolinol have been detected in the urine of Parkinson’s patients; thus, it has been speculated thar it contributes to the pathophysiology of Parkinson’s disease and chronic alcoholism. It has been demonstrated that when about 50–60% of the dopamine-producing nerve cells are lost, the symptoms of Parkinson’s disease begin to manifest. In animal studies, chronic administration of salsolinol induced parkinsonian-like symptoms. Moreover, little is known about its effects on gut–brain axis activation, with a possible neuroprotective property of this chemical compound also hypothesized [12]. Moreover, Wen et al., 2019 [54], reported that salsolinol reduces doxorubicin-induced chronic heart failure, reduces serum myocardial injury marker levels, decreases tissue damage to the heart, and increases the relative mRNA expression levels of key enzymes downstream of the TCA cycle that increase cardiac energy metabolism. However, it should be better clarified whether exogenous salsolinol has the same effects of salsolinol synthetized physiologically in animal organisms.

Polyamines such as SPM and SPD were also detected but only in a few samples, with maximum concentrations of about 44 mg kg^−1^_DDW_ and 106 mg kg^−1^_DDW_, respectively. Spermidine and spermine are naturally present in food [55]; in particular, SPD is most abundant in plant-based products, whilst SPM is generally higher in animal-derived foods [56,57]. Polyamine intake has important implications for human health [58], mainly for the intestinal and immune systems. Due to their antioxidant and anti-inflammatory effects, they are also important in the prevention of chronic diseases such as cardiovascular diseases. There are no recommendations for the daily intake of polyamines; however, dietary sources of polyamines become of greater importance in the aging population because the de novo synthesis of polyamines tends to decrease with age [59].

In Table 6, data are shown for the BAs in all the investigated samples, collected for three subclasses. In general, observing these results, it is evident that a significant positive effect in terms of a reduction in biogenic amines is seen in cocoa powder from less processed raw matter (organically grown cocoa, non-roasted cocoa beans).

Cocoa powder made from conventional process (not organic) showed higher levels of HIS and other BAs, so their sum was also significantly higher with respect to the levels in the organic samples. These results agree with the literature. Some authors have highlighted BAs as quality descriptors in cocoa products [42] and even as useful for differentiating between conventionally and organically grown cocoa [60,61]. They have identified cadaverine, serotonin, histamine, spermidine, spermine, tyramine, putrescine, and β-phenylethylamine, showing that organic samples contain lower concentrations of all of these amines.

In all the cocoa powder samples (N, 9) from raw cocoa beans, BAs were not detectable, whilst higher amounts for every single amine were detected in the samples from roasted beans.

Histamine being found in samples may result from the thermal decarboxylation of histidine during cocoa bean roasting [62]. Delgado-Ospina et al., 2020 [10], found a direct influence of different roasting temperatures on an increase in BAs, especially histamine and some polyamines, while a decrease in serotonin was found in dried roasted seeds. As pointed out by the authors, these values were not hazardous for human consumption.

As shown in Table 6, the alkalization process generally does not affect the presence of BAs, except for SAL and SPD, which were found only in non-alkalized samples. We can speculate that the results are probably correlated with the cocoa origin, which is very varied for some big trade farms.

### 3.5. Principal Component Analysis

Principal component analysis was carried out to obtain a representation of the numeric variables in a space of reduced dimensionality. The dimensionality of this space was chosen based on the portion of variance explained by each component. Based on graphical analysis of the screen plot, the four components chosen explained 31.2% (PC1), 18.8% (PC2), 14.7% (PC3), and 9% (PC4) of the variance, respectively. Through observation of the trend of square cosine (Cos2), it was possible to deduce that all the variables contribute to each component (quality of representation).

**Figure 1:**
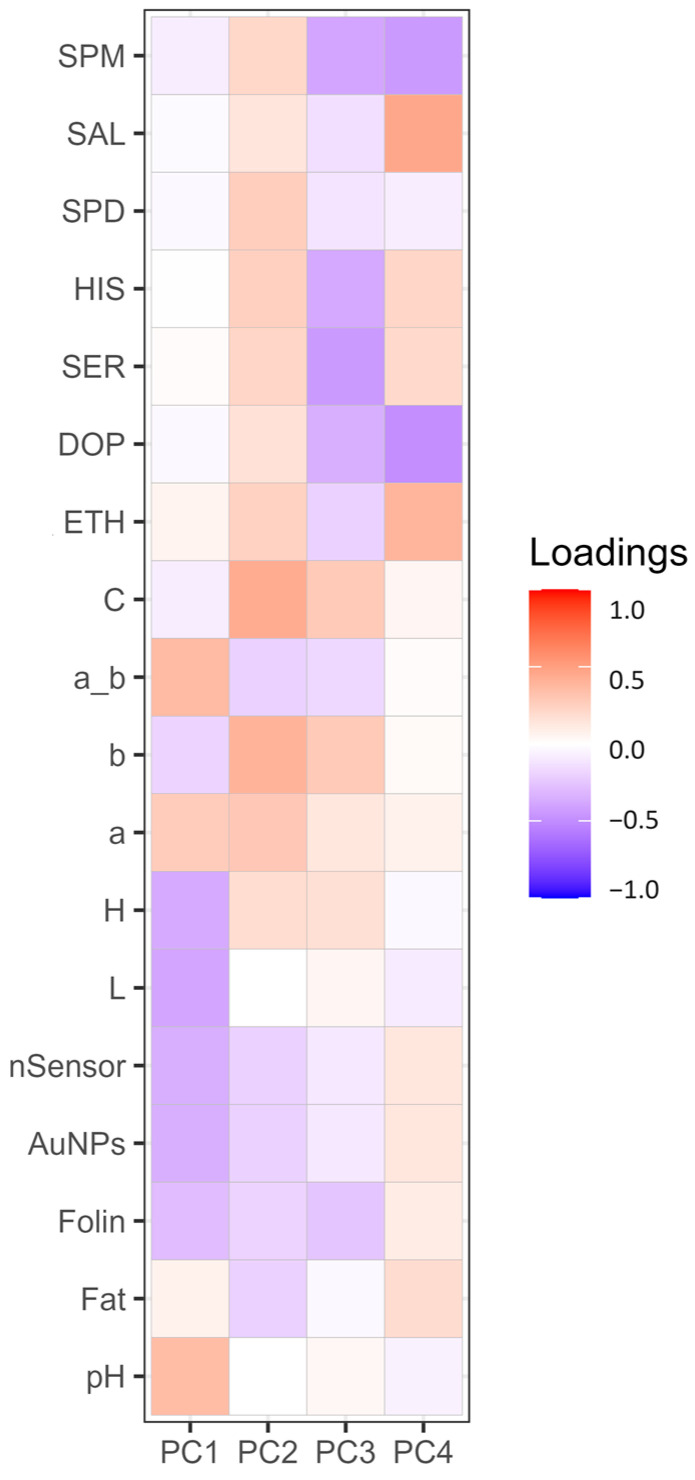
Heat map diagram displaying the loadings of variables for principal components PC1, PC2, PC3, and PC4. Different intensities are shown on the far right-hand side: deep blue and red coloration represents extremes of low and high intensity, respectively. SPM (spermine), SPD (spermidine), HIS (histamine), SER (serotonin), DOP (dopamine), ETH (ethanolamine), C (Chroma), a_b (ratio of colorimetric coordinates a* and b*), b (b* blueness/yellowness), a (a* redness/greenness), H (hue angle), L (lightness), nSensor (electrochemical nanostructured sensor), AuNPs (gold nanoparticle-based sensor).

**Figure 2:**
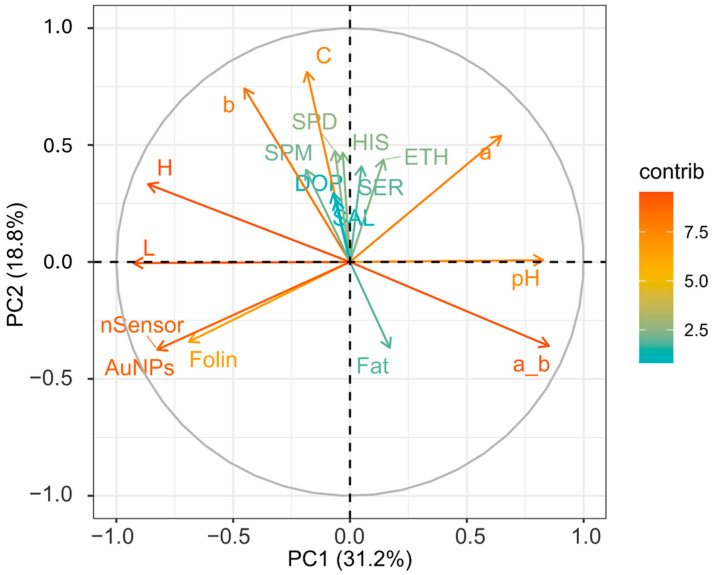
Loading plot of variables for the first two components. Different contributions are shown on the far right-hand side: deep green and orange coloration represents extremes of low and high contributions, respectively. SPM (spermine), SPD (spermidine), HIS (histamine), SER (serotonin), DOP (dopamine), ETH (ethanolamine), C (Chroma), a_b (ratio of colorimetric coordinates a* and b*), b (b*blueness/yellowness), a (a* redness/greenness), H (hue angle), L (lightness), nSensor (electrochemical nanostructured sensor), AuNPs (gold nanoparticle-based sensor).

Figure 1 represents a heat map showing the loadings of each variable for PC1, PC2, PC3, and PC4. PCA was performed to highlight how the factors or variables could distinguish between cocoa powder samples obtained with different technological processes. Figure 2 shows graphically the loadings for PC1 and PC2.

From Figure 1, it is visible how for PC1 and PC2, the different loadings impact on distinguishing between the samples. PC1 sees two extremes for pH and L; this separation somehow reflects the nature of the cocoa powders, informing us about the effect of the alkalinization process [63]. This, among all the changes, alters the pH and the color. As is visible, the pH variable influences the ratio a*/b*, which has a similar positive contribution (Figure 1) and spatial localization in the PCA plot (Figure 2), with pH (0.34) and red index a*/b* (0.36) showing a positive effect. On the other hand, L (lightness) has a negative contribution (Figure 1), localized in the opposite region of the PCA plot (Figure 2) (L) (−0.39). Of course, as for the pH’s influence on the a*_b* ratio, L correlates with H (hue angle) (−0.36); this property describes the color position on a color wheel expressed in degrees ° or according to its main wavelength. Thus, even if the color itself does not change, differences in L* increment or diminish the color intensity, making it possible to distinguish between lighter or darker shades. Alkalized products tend to have higher pH values and be darker shades of brown. This is in line with the general market demand for cocoa and chocolate products despite the recent introduction of ruby chocolate and related products [64,65]. Polyphenols, evaluated by the nSensor and the AuNP and Folin assays, all negatively contribute to PC1 (Figure 1); the spatial positioning on the PCA plot attests to these three variables greatly contributing and highly demarcating the samples distributed around the variables Folin (−0.29) and AuNP-based assay (−0.34). Once again, technological treatment impacts the final content of flavanols and other phenolic species as measured with different indexes. As attested to, alkalization reduces bitterness by limiting the presence of polyphenols and increasing the rate of Maillard reactions of conjugation among sugars and amino acids [18]. Concerning PC2, this component was mainly influenced by the colorimetric parameters. The second component was associated with increasing C*, b*, and a*. PC2 also receives a positive contribution from biogenic amines and salsolinol, as visible from Figure 1. Regardless, the PCA explains better how their role is limited. The less discriminative power of the BAs and salsolinol in the PCA tells us that these compounds are not influenced by the alkali process, as happens for the colorimetric indexes. From these analyses, it can be stated that the technological process, namely alkalization, changes many features of cocoa powders, as well as acting on composition traits (pH, color, and phenolic composition), but with a limited extension to the evolution of certain constitutive compounds (BAs and salsolinol), which are more dependent on the obtention of raw matter and other previous technological process, such as fermentation and roasting.

**Figure 3:**
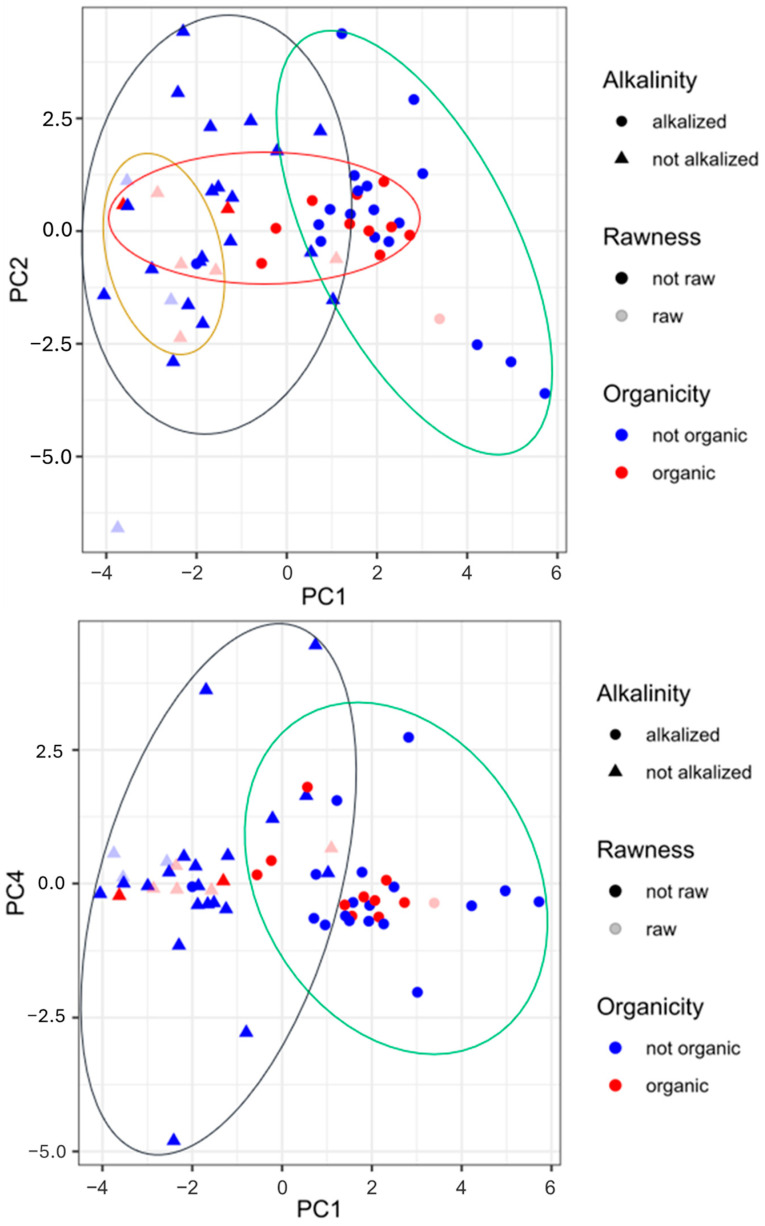
Score plot obtained by principal component analysis of pH, colorimetric parameters, biogenic amine content, and polyphenol content in cocoa powder samples under different technological treatments. Legend: circle, alkalized cocoa powder; triangle, non-alkalized samples; blue circle, non-organic cocoa powder; red circle, organic samples; light blue symbol, cocoa powder from roasted beans; light red symbol, cocoa samples from raw beans.

Figure 3 completes the information given in the heat map and the PCA. The graphs in Figure 3 show score plots for PC1 and PC2 and for PC1 and PC4, respectively. As visible from the first score plot, there is a strong separation between the alkalized and non-alkalized samples, highly spread for PC1. By overlapping the variable distribution (Figure 2), it is clear that pH and a*_b* ratio are discriminants for alkalized samples (on the right side of the plots), while L and H, b*, and C* are discriminants for non-alkalized samples (these have more vivid colors and are lighter). The samples, mainly blue circles, at the very far right side of the plot may be powders for which the extension of the Dutch process was longer or for which this process was conducted with different reagents (i.e., NaHCO_3_, K_2_CO_3_, KOH) and more intense conditions (temperature and pressure). The obtention of cocoa, and thus organic or conventional farming, and its rawness do not have a great impact on the differentiation of the samples. Regardless, in the first plot, all of the organic samples and most of the powders obtained from raw beans, no matter the technological processes employed, may be grouped. The second score plot illustrates again the marked difference between the non-alkalized and alkalized samples, spread in the horizontal direction (PC1). The other traits (organicity and rawness) are not potent enough to distinguish the samples.

## 4. Conclusions

This survey offers vast insights into the qualitative state of marketed cocoa powders. Several compounds were examined, and multiple differences in the technological processes and agronomical practices for cocoa production were considered. For the samples described here, the technological process of alkalinization (or the Dutch process) caused the main changes, highly separating the treated and untreated powders.

As is known, cocoa is alkalized to improve cocoa powder’s solubility while reducing its bitterness and acidity and giving commercial powders their typical color and aroma. In fact, for a long time, these characteristics have driven consumer choices for this product. Alkalinization, on the one hand, had a tremendous impact on the phenolic fraction, reducing it, and, on the other hand, did not affect the presence and/or evolution of certain compounds, such as biogenic amines and salsolinol.

Even if all the samples may be considered safe (with respect to the content of the latter compounds), we registered samples positive for histamine, known for its vasoactive effects and toxicity. Concern lies in the fact that the available cocoa powders are often a blend of cocoa from different producers, possibly mixing powders high in biogenic amines and salsolinol-positive. This may pose a safety issue given that the toxicity of a single biogenic amine may be enhanced by the activity of others, especially for those subjects with a specific sensitivity or whose detoxifying systems are overloaded for other reasons. As concerns the phenolic fraction, this paper offers a direct comparison of different methods, including the most common, the Folin–Ciocalteu assay, a gold nanoparticle-based assay, and an electrochemical nanostructured sensor.

As the ultimate conclusion, it can be affirmed that this work may be a starting point for deepening the topic of quality evaluation of cocoa powders and related products, as well as presenting innovative methods and uncovering less investigated active compounds, such as salsolinol, on which more research is needed.

## Figures and Tables

**Table 1:** Geographic origin of the samples sourced from the European market.

Provider Code (N of Samples Collected)	Number Code	Geographic Origin	Alkalinization	Raw Samples	Organic Samples
BC (N = 15)	1; 5; 8; 12–14; 38–46	Ecuador; Sao Tomè; Dominican Republic; Peru	4, 10, 13, 14, 17–24, 30, 31, 34, 36, 37, 40–44, 46–48, 50–53	1–3, 5, 26, 27, 53, 58, 59	2, 3, 6, 13, 14, 17, 19, 26, 27, 35, 36, 37, 48, 50, 51, 53
CA (N = 1)	6	Colombia			
CE (N = 2)	4; 10	Ivory Coast			
CG (N = 8)	7; 16; 19; 21–25	Ivory Coast			
CM (N = 1)	15	Ivory Coast			
DN (N = 5)	11; 28–31	Ghana			
IM (N = 13)	17; 18; 20; 32–35; 36;37; 47–48; 50–51	Ecuador; Peru			
JR (N = 3)	26–27; 53	Ecuador			
ME (N = 2)	58–59	Bali			
PD (N = 6)	9; 49; 54–57	Vietnam			
UO (N = 1)	3	Colombia			
VV (N = 1)	2	Bali			
ZI (N = 1)	52	Ivory Coast			

**Table 2:** Technological parameters (means ± standard deviation; values in brackets are min and max values) of cocoa powder samples belonging to different categories.

	L*	a*	b*	C	h°	pH
not organic (N. 41)	41.49 ± 7.50	12.97 ± 1.63	19.59 ± 3.19	23.58 ± 3.02	56.17 ± 4.99	6.02 ± 0.78
	(24.77–55.61)	(9.05–16.35)	(10.95–25.64)	(14.20–28.67)	(41.29–63.63)	(4.75–7.32)
organic (N. 18)	40.71 ± 6.08	13.45 ± 1.46	20.16 ± 2.20	24.30 ± 2.00	56.19 ± 4.09	6.38 ± 0.84
	(30.08–52.09)	(10.93–15.69)	(14.86–24.05)	(20.02–27.27)	(47.96–64.79)	(5.08–7.32)
*p*-value	0.675	0.320	0.499	0.363	0.985	0.121
not raw (N. 50)	40.32 ± 7.29	19.34 ± 1.54	19.87 ± 2.61	24.00 ± 2.42	55.93 ± 4.62	6.24 ± 0.81
	(24.77–55.61)	(9.71–16.35)	(11.17–24.12)	(16.93–27.61)	(41.29–64.79)	(4.75–7.32)
raw (N. 9)	46.46 ± 5.39	11.92 ± 1.30	19.22 ± 4.43	22.69 ± 4.18	57.50 ± 5.19	5.55 ± 0.58
	(33.17–51.25)	(9.05–13.41)	(10.95–25.64)	(14.20–28.67)	(47.96–63.41)	(5.08–6.87)
*p*-value	0.019	0.012	0.716	0.238	0.246	0.009
not alkalized (N. 30)	46.52 ± 5.52	12.18 ± 1.42	20.51 ± 2.92	23.89 ± 2.95	59.11 ± 3.39	5.39 ± 0.28
	(32.86–55.61)	(9.05–14.82)	(10.95–25.64)	(14.20–28.67)	(50.46–64.79)	(4.75–5.95)
alkalized (N. 29)	35.80 ± 4.44	14.10 ± 1.06	19.00 ± 2.76	23.70 ± 2.58	53.14 ± 3.87	6.90 ± 0.28
	(24.77–42.23)	(11.86–16.35)	(11.17–22.35)	(16.93–27.53)	(41.29–57.93)	(6.01–7.32)
*p*-value	<0.001	<0.001	0.055	0.806	<0.001	<0.001

**Table 3:** Technological sample features; fat and phenolic compound evaluation; data are expressed as mean values (n = 3) and standard deviation.

		Total Phenolic Content (mg_GAeq_ g^−1^)		
Sample	Fat (%)	Folin	AuNPs	nSensor
1	5.6 ± 0.9	141.0 ± 3.7	136.9 ± 7.1	57.1 ± 0.4
2	15.1 ± 1.3	72.0 ± 1.4	79.1 ± 0.5	33.0 ± 0.9
3	8.5 ± 0.7	60.8 ± 1.6	51.7 ± 0.2	21.6 ± 0.4
4	24.6 ± 2.1	36.2 ± 2.3	43.8 ± 0.1	18.3 ± 0.8
5	7.0 ± 0.6	51.8 ± 1.1	71.0 ± 0.8	29.6 ± 0.8
6	8.5 ± 0.7	67.8 ± 1.0	52.0 ± 6.1	21.7 ± 2.0
7	9.3 ± 1.2	48.1 ± 0.2	70.1 ± 7.2	29.3 ± 0.6
8	9.1 ± 1.8	73.7 ± 1.2	39.5 ± 6.0	16.5 ± 2.2
9	9.1 ± 0.8	53.5 ± 0.7	72.9 ± 2.3	30.5 ± 1.8
10	20.7 ± 1.8	35.7 ± 1.6	39.0 ± 2.1	16.3 ± 1.7
11	7.9 ± 0.7	31.1 ± 2.4	39.4 ± 1.5	16.5 ± 0.8
12	8.8 ± 0.7	24.9 ± 3.0	31.9 ± 0.6	13.4 ± 1.5
13	10.4 ± 0.9	15.8 ± 0.2	13.9 ± 0.1	5.9 ± 0.7
14	16.5 ± 1.4	14.2 ± 0.1	11.9 ± 0.2	5.1 ± 0.8
15	14.3 ± 1.2	20.3 ± 0.2	31.2 ± 0.9	13.1 ± 0.9
16	8.7 ± 0.7	25.3 ± 1.7	40.1 ± 1.1	16.8 ± 0.6
17	17.5 ± 1.5	20.1 ± 1.7	20.4 ± 1.0	8.6 ± 1.5
18	10.2 ± 0.9	25.9 ± 1.0	18.7 ± 0.9	7.9 ± 1.0
19	8.1 ± 0.7	9.2 ± 0.2	8.9 ± 0.3	3.8 ± 0.4
20	12.4 ± 1.1	22.3 ± 0.2	21.1 ± 0.4	8.9 ± 0.9
21	8.7 ± 0.9	16.0 ± 0.1	10.5 ± 0.2	4.5 ± 0.9
22	19.9 ± 1.8	14.0 ± 0.0	6.6 ± 0.1	2.8 ± 0.8
23	20.5 ± 1.7	9.5 ± 0.0	8.6 ± 0.5	3.7 ± 0.4
24	11.5 ± 1.0	8.8 ± 0.1	4.5 ± 0.2	2.0 ± 0.7
25	22.1 ± 1.9	33.3 ± 0.0	38.2 ± 5.2	16.0 ± 1.3
26	9.7 ± 0.8	43.3 ± 0.3	63.9 ± 1.7	26.7 ± 1.7
27	20.9 ± 1.8	32.8 ± 0.6	38.1 ± 0.9	15.9 ± 1.1
28	9.0 ± 0.8	37.6 ± 2.7	48.9 ± 2.2	20.5 ± 2.3
29	20.7 ± 1.8	27.3 ± 0.4	32.2 ± 0.3	13.5 ± 1.3
30	7.9 ± 0.7	3.6 ± 0.4	13.5 ± 0.1	5.7± 0.8
31	7.5 ± 0.6	16.9 ± 0.0	9.9± 0.4	4.2 ± 0.6
32	9.3 ± 0.8	43.4 ± 1.2	49.2 ± 0.1	20.6 ± 0.8
33	10.3 ± 0.9	44.2 ± 1.6	48.0 ± 1.1	20.1 ± 0.2
34	6.4 ± 0.5	33.2 ± 0.0	12.4 ± 0.0	5.2 ± 0.1
35	17.9 ± 1.5	39.9 ± 0.9	38.3 ± 1.9	16.0 ± 0.5
36	9.6 ± 0.8	45.2 ± 2.1	59.4 ± 1.4	24.8± 1.6
37	15.9 ± 1.4	49.8 ± 2.0	45.7 ± 0.4	19.1 ± 1.2
38	9.5 ± 0.8	40.2 ± 1.9	50.2 ± 0.4	21.0 ± 1.8
39	20.7 ± 1.8	67.6 ± 0.5	49.7 ± 5.2	20.8 ± 0.7
40	8.0 ± 0.9	21.1 ± 0.2	7.2 ± 0.3	3.1 ± 0.6
41	19.3 ± 1.6	22.5 ± 0.2	16.4 ± 0.2	6.9 ± 0.1
42	8.9 ± 0.8	17.1 ± 0.1	9.6 ± 0.3	4.1 ± 0.3
43	8.2 ± 0.6	30.9 ± 2.0	14.8 ± 1.0	6.2 ± 0.9
44	7.6 ± 0.6	15.3 ± 0.2	10.9 ± 0.1	4.6 ± 0.6
45	9.3 ± 0.8	51.8 ± 1.5	55.5 ± 2.5	23.2 ± 0.2
46	7.3 ± 0.6	25.6 ± 1.9	35.5 ± 0.7	14.9 ± 0.5
47	21.0 ± 1.8	26.0 ± 0.5	21.8 ± 1.8	9.2 ± 1.1
48	16.6 ± 1.4	35.0 ± 1.2	30.7 ± 0.4	12.9 ± 0.3
49	27.3 ± 2.3	56.8 ± 2.0	63.8 ± 0.4	26.7 ± 2.1
50	17.5 ± 1.7	26.6 ± 2.6	14.6 ± 0.4	6.2 ± 1.2
51	17.4 ± 1.4	65.4 ± 1.8	58.2 ± 0.2	24.3 ± 1.6
52	18.8 ± 1.7	14.9 ± 0.2	8.8 ± 0.1	3.7 ± 0.3
53	17.1 ± 1.3	25.2 ± 1.5	13.3 ± 0.6	5.6 ± 0.8
54	22.1 ± 1.9	46.2 ± 0.5	45.9 ± 0.3	19.2 ± 1.5
55	26.1 ± 2.2	64.3 ± 0.7	59.7 ± 0.3	24.9 ± 0.7
56	23.5 ± 2.0	37.0 ± 0.4	42.5 ± 2.4	17.8 ± 0.8
57	18.9 ± 1.6	28.7 ± 1.1	63.6 ± 1.6	26.6 ± 2.1
58	16.2 ± 1.4	69.7 ± 0.1	71.5 ± 1.2	29.8 ± 2.1
59	11.2 ± 1.0	50.4 ± 0.5	68.8 ± 2.0	28.7 ± 0.8

**Table 4:** Phenolic compounds (means ± standard deviation; values in brackets are min and max values) of cocoa powder samples belonging to different categories.

	Phenolic Compounds (mg_GAEeq_ g^−1^_DDW_)	
	TPC	nSensor
conventional (N. 41)	42.18 ± 27.45	18.13 ± 12.57
	(3.94–149.2)	(2.21–60.47)
organic (N. 18)	45.39 ± 22.27	19.21 ± 10.51
	(9.97–84.71)	(4.11–38.86)
*p*-*value*	*0.467*	*0.477*
not raw (N. 50)	38.61 ± 21.19	16.20 ± 9.91
	(3.94–86.99)	(2.21–36.69)
raw (N. 9)	68.44 ± 35.19	31.03 ± 14.64
	(30.40–149.2)	(6.77–60.47)
*p*-*value*	*0.003*	*0.003*
not alkalized (N. 30)	57.48 ± 25.38	26.57 ± 9.32
	(23.67–149.2)	(14.65–60.47)
alkalized (N. 29)	28.35 ± 16.38	10.07 ± 7.73
	(3.94–79.23)	(2.21–29.44)
*p*-*value*	*<0.001*	*<0.001*

**Table 5:** Results of biogenic amine analysis in cocoa powder sourced from the European market (N = 59).

	ETH	DOP	SER	HIS	SPD	SAL	SPM	Total BAs
N of positive (%)	9 (15%)	3 (5%)	13 (22%)	11 (19%)	5 (8%)	4 (7%)	3 (5%)	18 (31%)
*BAs* (mg kg^−1^_DDW_)								
median	0.00	0.00	0.00	0.00	0.00	0.00	0.00	0.00
median (of positive *samples)*	22.89	88.32	82.13	69.70	23.88	0.22	25.35	154.08
mean	6.11	5.80	18.53	11.40	3.53	0.06	1.46	46.89
dev st	20.23	27.23	38.97	28.21	15.92	0.31	6.84	95.07
minimum	0.00	0.00	0.00	0.00	0.00	0.00	0.00	0.00
maximum	91.54	176.95	181.81	139.28	105.96	1.86	43.66	480.87

**Table 6:** Biogenic amine (mg kg^−1^_DDW_) levels in the subclasses; data reported as means ± standard deviation; values in brackets are min and max values.

	ETH	DOP	SER	HIS	SPD	SAL	SPM	Total BAs
conventional (N. 41)	7.15 ± 22.05	8.34 ± 32.46	23.54 ± 43.42	15.75 ± 32.76	5.08 ± 18.96	0.09 ± 0.37	2.10 ± 8.15	62.04 ± 107.95
	(0.00–91.54)	(0.00–176.95)	(0.00–181.81)	(0.00–139.28)	(0.00–105.96)	(0.00–1.86)	(0.00–43.66)	(0.00–480.87)
organic (N. 18)	3.76 ± 15.61	0.00 ± 0.00	7.14 ± 23.40	1.49 ± 6.33	0.00 ± 0.00	0.00 ± 0.00	0.00 ± 0.00	12.39 ± 39.99
	(0.00–66.28)	(0.00–0.00)	(0.00–95.62)	(0.00–26.84)	(0.00–0.00)	(0.00–0.00)	(0.00–0.00)	(0.00–161.90)
*p*-*value*	0.464	0.455	0.573	0.158	0.199	0.107	0.174	0.832
not raw (N. 50)	7.21 ± 21.82	6.84 ± 29.51	21.87 ± 41.51	13.45 ± 30.23	4.16 ± 17.24	0.07 ± 0.34	1.72 ± 7.41	55.33 ± 101.10
	(0.00–91.54)	(0.00–176.95)	(0.00–181.81)	(0.00–139.28)	(0.00–105.96)	(0.00–1.86)	(0.00–43.66)	(0.00–480.87)
raw (N. 9)	0.00 ± 0.00	0.00 ± 0.00	0.00 ± 0.00	0.00 ± 0.00	0.00 ± 0.00	0.00 ± 0.00	0.00 ± 0.00	0.00 ± 0.00
	(0.00–0.00)	(0.00–0.00)	(0.00–0.00)	(0.00–0.00)	(0.00–0.00)	(0.00–0.00)	(0.00–0.00)	(0.00–0.00)
*p*-*value*	0.246	0.152	0.372	0.174	0.461	0.838	0.178	0.159
not alkalized (N. 30)	3.82 ± 13.39	8.84 ± 35.61	19.93 ± 44.54	15.70 ± 29.40	4.42 ± 19.67	0.12 ± 0.43	2.87 ± 9.46	55.71 ± 108.08
	(0.00–69.61)	(0.00–176.95)	(0.00–181.81)	(0.00–86.25)	(0.00–105.96)	(0.00–1.86)	(0.00–43.66)	(0.00–480.87)
alkalized (N. 29)	8.48 ± 25.50	2.65 ± 14.25	17.08 ± 32.96	6.95 ± 26.71	2.61 ± 11.08	0.00 ± 0.02	0.00 ± 0.00	37.77 ± 80.33
	(0.00–91.54)	(0.00–76.73)	(0.00–107.60)	(0.00–139.28)	(0.00–57.48)	(0.00–0.08)	(0.00–0.02)	(0.00–296.41)
*p*-*value*	0.399	0.781	0.425	0.161	0.686	0.171	0.175	0.380

## Data Availability

The data presented in this study are available on request from the corresponding author. The data are unavailable due to privacy.
